# *Lactiplantibacillus plantarum* NKK20 Alleviates High-Fat-Diet-Induced Nonalcoholic Fatty Liver Disease in Mice through Regulating Bile Acid Anabolism

**DOI:** 10.3390/molecules28104042

**Published:** 2023-05-12

**Authors:** Chang Sun, Chenguang Qiu, Yanyan Zhang, Man Yan, Jiajun Tan, Jiayuan He, Dakai Yang, Dongxu Wang, Liang Wu

**Affiliations:** 1 Department of Laboratory Medicine, School of Medicine, Jiangsu University, Zhenjiang 212013, China; 2 Department of Stomatology, Zhenjiang First People’s Hospital, Zhenjiang 212002, China; d.charlie@163.com; 3 Department of Testing Center, Yangzhou University, Yangzhou 225001, China; 4 Zhenjiang Center for Disease Control and Prevention, Zhenjiang 212002, China; 5 School of Grain Science and Technology, Jiangsu University of Science and Technology, Zhenjiang 212100, China; 6 Department of Laboratory Medicine, Lianyungang Second People’s Hospital Affiliated to Jiangsu University, Lianyungang 222006, China

**Keywords:** *Lactiplantibacillus plantarum*, NAFLD, bile acid anabolism, inflammation, probiotics, metabolomics

## Abstract

Nonalcoholic fatty liver disease (NAFLD) is the most prevalent chronic disease in modern society. It is characterized by an accumulation of lipids in the liver and an excessive inflammatory response. Clinical trials have provided evidence that probiotics may prevent the onset and relapse of NAFLD. The aim of this study was to explore the effect of *Lactiplantibacillus plantarum* NKK20 strain (NKK20) on high-fat-diet-induced NAFLD in an ICR murine model and propose the underlying mechanism whereby NKK20 protects against NAFLD. The results showed that the administration of NKK20 ameliorated hepatocyte fatty degeneration, reduced total cholesterol and triglyceride concentrations, and alleviated inflammatory reactions in NAFLD mice. In addition, the 16S rRNA sequencing results indicated that NKK20 could decrease the abundance of *Pseudomonas* and *Turicibacter* and increase the abundance of *Akkermansia* in NAFLD mice. LC-MS/MS analysis showed that NKK20 could significantly increase the concentration of short-chain fatty acids (SCFAs) in the colon contents of mice. The obtained non-targeted metabolomics results revealed a significant difference between the metabolites in the colon contents of the NKK20 administration group and those in the high-fat diet group, in which a total of 11 different metabolites that were significantly affected by NKK20 were observed, and these metabolites were mainly involved in bile acid anabolism. UPLC-MS technical analysis revealed that NKK20 could change the concentrations of six conjugated and free bile acids in mouse liver. After being treated with NKK20, the concentrations of cholic acid, glycinocholic acid, and glycinodeoxycholic acid in livers of the NAFLD mice were significantly decreased, while the concentration of aminodeoxycholic acid was significantly increased. Thus, our findings indicate that NKK20 can regulate bile acid anabolism and promote the production of SCFA, which can inhibit inflammation and liver damage and thus prevent the development of NAFLD.

## 1. Introduction

With the rapid development of the social economy, the corresponding unhealthy lifestyles have provoked increased rates of obesity and metabolic syndrome globally, which, in turn, contributes to increasing the prevalence of nonalcoholic fatty liver disease (NAFLD) [1]. In essence, NAFLD is a clinicopathological syndrome characterized by the dysfunction of fat metabolism and the excessive deposition of large amounts of fat in liver cells [2]. Moreover, NAFLD can lead to cardiovascular disease (such as atherosclerosis and coronary heart disease) and endocrine diseases (such as type 2 diabetes) by aggravating metabolic disorders of the body and increasing the risk of chronic kidney disease, colon cancer, liver cancer, osteoporosis, and other extrahepatic diseases [3,4]. With an increasing elderly population, China has the fastest-growing prevalence rate of NAFLD in the world; at present, the prevalence rate is about 29.2%, and the number of NAFLD patients will further increase rapidly in the future [5].

Although the pathogenesis of NAFLD is still not fully understood, the “second strike” theory, which proposes that the accumulation of lipids in the liver and chronic inflammation contribute to NAFLD development, has been widely recognized [6]. Obesity, insulin resistance, and other factors, together constituting a “first blow”, lead to the accumulation of lipids in the liver and even the formation of non-alcoholic simple fatty liver disease; however, non-alcoholic steatohepatitis occurs when the liver is damaged by inflammation and oxidative stress due to a “second shock” [7,8]. In order to treat NAFLD effectively, lipid metabolism disorders must be corrected, and the inflammatory response must be suppressed [9]. However, there is still a lack of a safe and effective treatment that lowers blood lipid levels and reduces hepatic inflammation [10].

It is believed that probiotics can provide health benefits when supplemented appropriately. Studies have proven that the oral administration of a variety of *Lactiplantibacillus* species and their products can effectively reduce triglyceride (TG) and total cholesterol (TC) levels in serum and reduce the potency of the systemic inflammatory response, which can be used in the adjuvant treatment of NAFLD and other diseases, but the exact mechanism of this activity is not yet understood [11,12]. *Lactiplantibacillus plantarum* NKK20 strain (NKK20) is a newly isolated strain from the intestines of healthy humans that has been deposited in the China Typical Culture Preservation Center under the preservation number CCTCC NO: M2020596. This NKK20 strain has strong acid resistance, bile salt resistance, and intestinal mucosal cell adhesion abilities and confers strong hypolipidemic and anti-inflammatory effects [13,14]. The objective of this study was to use metabolomics to examine the mechanism by which *L. plantarum* NKK20 reduces blood-lipid-related indices and inhibits inflammation in mice with NAFLD. The present study provides key insights into the way *L. plantarum* NKK20 alleviates NAFLD via the “intestinal flora-metabolites-inflammation” axis.

## 2. Results

### 2.1. Influence of NKK20 on Body Weight and Blood Lipids in NAFLD Mice

**Figure 1:**
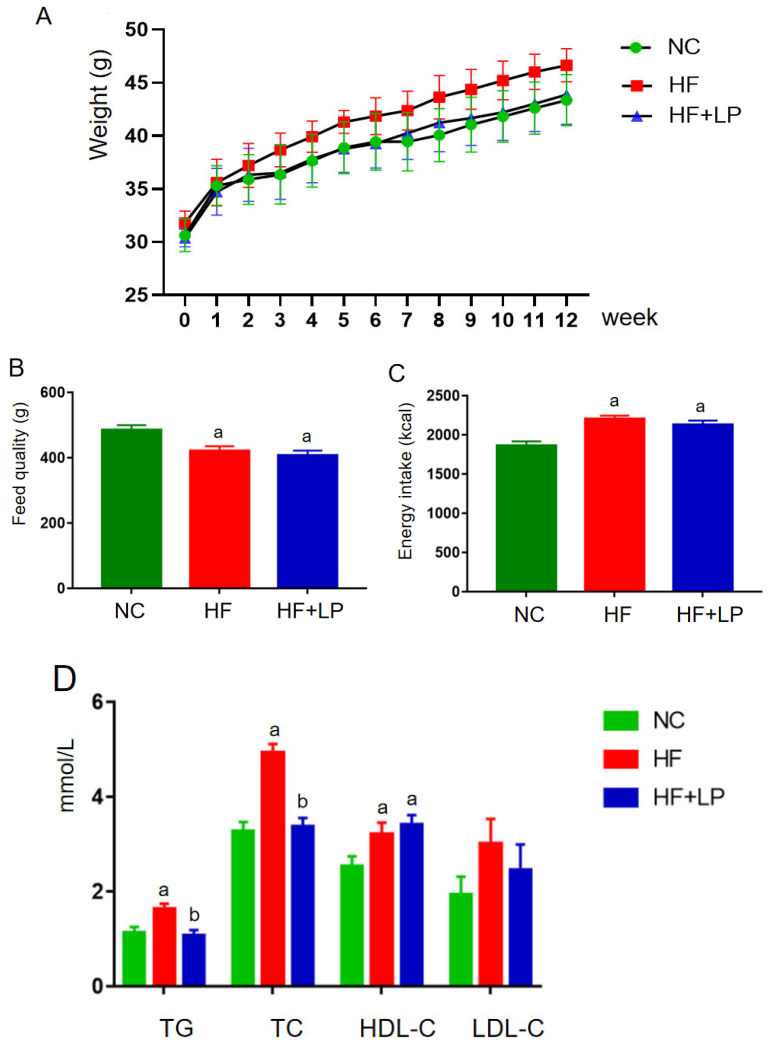
The change in body weight and lipid-related indices in mice: (**A**) body weight; (**B**) feed quality; (**C**) energy intake (kcal); (**D**) plasma concentrations of lipid-related indices. Data are presented as means ± SD (n = 10). a: Compared with the NC group, *p* < 0.05; b: compared with the HF group, *p* < 0.05.

During the entire experimental period, the body weight of each group showed an increasing trend (Figure 1A). In the second week, the body weight of the mice in the HF group was higher than that in the NC and HF + LP groups, and this trend continued until the end of the experiment. Starting in the sixth week, the body weight of the mice in the HF + LP group was higher than that in the NC group but still lower than that in the HF group, and this trend continued until the end of the experiment. Throughout the whole experiment, the feed intake in the NC group was higher than that in the HF and HF + LP groups, but energy intake was lower than that in the HF and the HF + LP groups (*p* < 0.05), and there was no statistical significance between the HF group and HF + LP group with respect to energy intake (*p* > 0.05) (Figure 1B,C). At the end of the experiment, the concentrations of TC, TG, and HDL-C in the HF and HF + LP groups were significantly higher than those in the NC group (*p* < 0.05), while the concentration of LDL-C showed no significant changes (*p* > 0.05). Compared with the HF group, the concentrations of TC and TG in the HF + LP group were significantly decreased (*p* < 0.05), but there were no significant differences in the concentrations of HDL-C and LDL-C (*p* > 0.05) (Figure 1D).

### 2.2. Influence of NKK20 on Splenic/Hepatic Inflammatory Factors and Hepatic Cholesterol-Metabolizing Enzymes’ Expression and Intestinal Microbiota in NAFLD Mice

**Figure 2:**
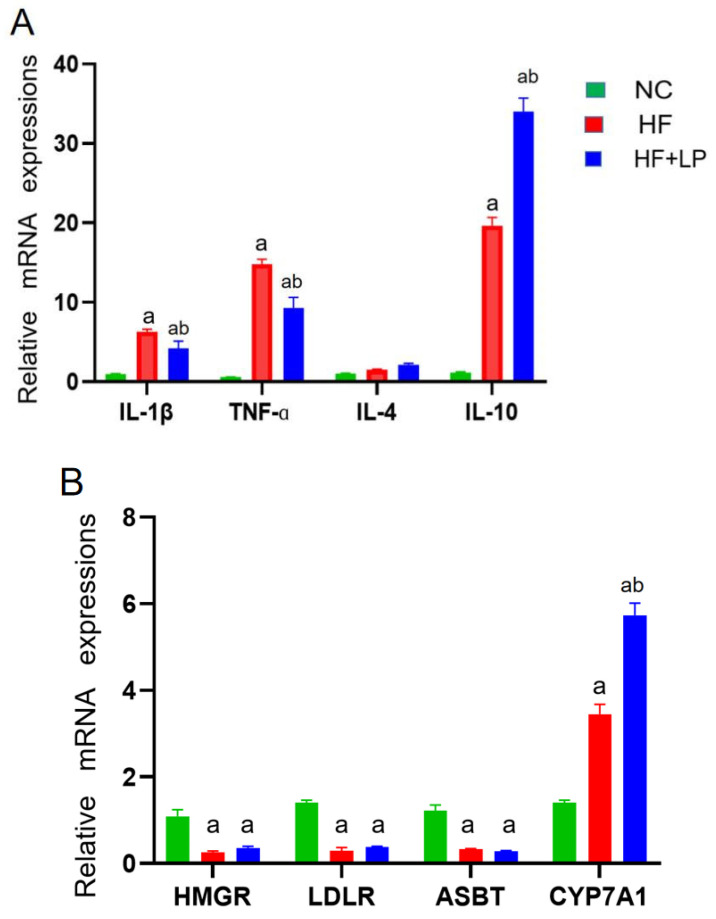

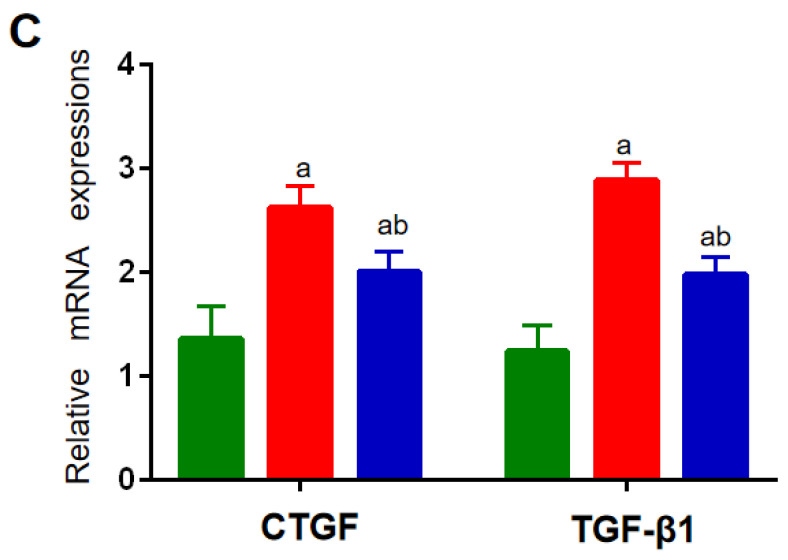
The change in splenic-hepatic inflammatory factors and hepatic cholesterol-metabolizing enzymes’ expression levels and intestinal microbiota in NAFLD mice. (**A**) The mRNA expression of inflammatory factors in the spleen. (**B**) mRNA expression of cholesterol-metabolizing enzymes in the liver. (**C**) The mRNA expression of liver-fibrosis-related indicators. Data are presented as the mean ± SD (n = 10). a: Compared with the NC group, *p* < 0.05; b: compared with the HF group, *p* < 0.05.

The expression levels of the factors related to inflammatory and cholesterol-metabolizing enzymes were determined using a qRT-PCR assay. Compared with the NC group, the mRNA expressions of IL-1β, TNF-α, and IL-10 in the spleen in the HF and HF + LP groups were significantly increased (*p* < 0.05), while the mRNA expression of IL-4 was not significantly changed (*p* > 0.05) (Figure 2A). Compared with the HF group, the mRNA expression levels of IL-1β and TNF-α in the spleen in the HF-LP group were significantly decreased (*p* < 0.05), while the mRNA expression of IL-10 was significantly increased (*p* < 0.05) and the mRNA expression of IL-4 was not significantly changed (*p* > 0.05) (Figure 2A). Compared with the NC group, the mRNA expressions of 3-hydroxy-3-methylglutaryl coenzyme A reductase (HMGR), low-density lipoprotein receptor (LDLR), and bile acid transporter (ASBT) in the liver in the HF and HF + LP groups were significantly decreased (*p* < 0.05), while the mRNA expression of cholesterol 7a-hydroxylase (CYP7A1) was significantly increased (*p* < 0.05) (Figure 2B). Compared with the HF group, only the mRNA expression of CYP7A1 was significantly increased in the HF + LP group (*p* < 0.05), while other indicators had no significant changes (*p* > 0.05) (Figure 2B). Compared with the NC group, the mRNA expression levels of connective tissue growth factor (CTGF) and transforming growth factor-β1 (TGF-β1) in the liver in the HF and HF + LP groups were significantly increased (*p* < 0.05) (Figure 2C). Compared with the HF group, the mRNA expression levels of CTGF and TGF-β1 were significantly decreased in the HF + LP group (*p* < 0.05) (Figure 2C).

### 2.3. Influence of NKK20 on Liver Tissue in NAFLD Mice

**Figure 3:**
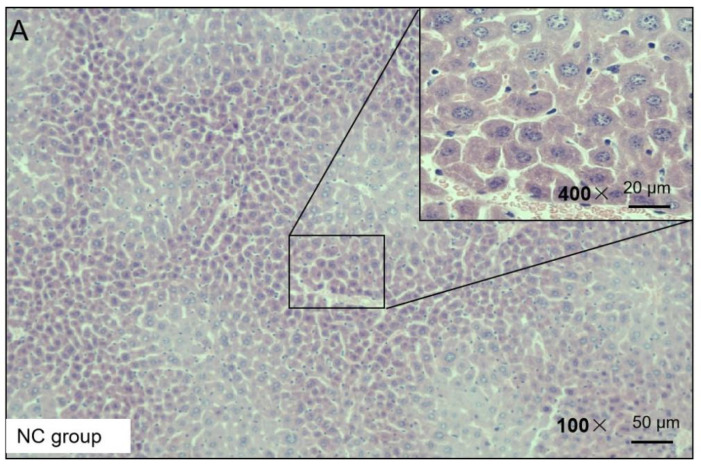

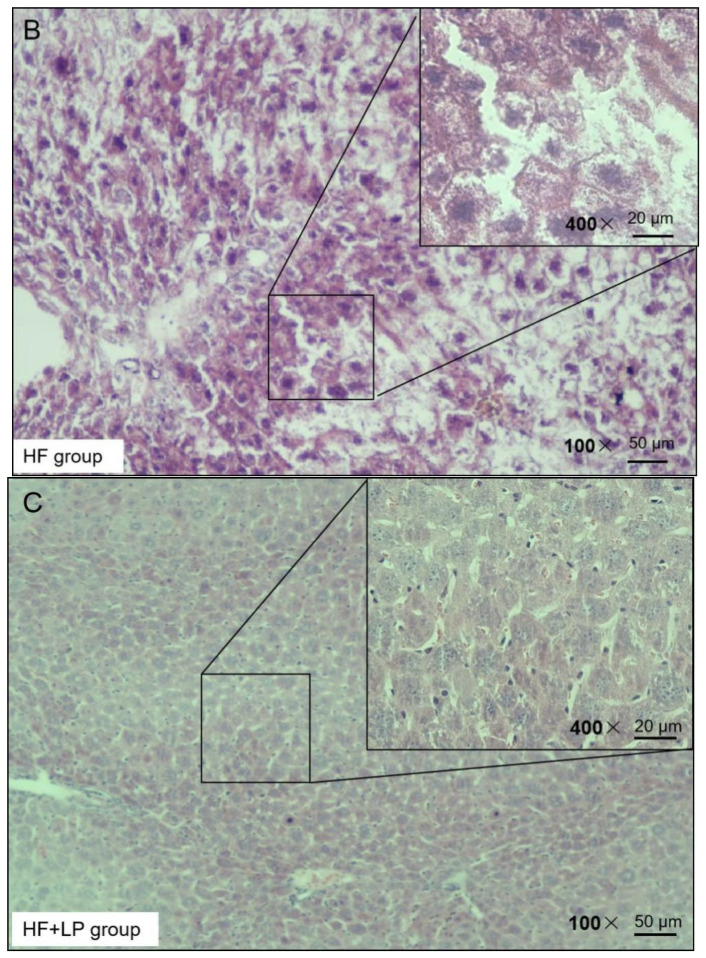
Representative H&E staining of mouse liver tissue.

H&E staining was used to observe the liver injury of the NAFLD mice. The H&E staining results presented the following findings: the morphology of the liver cells in the NC group was normal, no obvious degeneration or necrosis was observed, and no inflammatory cells or fiber tissue proliferation were observed in the portal area (Figure 3). In the HF group, the liver tissue was almost replaced by adipocyte vacuoles, and, concerning hepatocyte balloon-like degeneration, no fibrous tissue hyperplasia was observed (Figure 3). In the HF + LP group, the distribution of adipose vacuoles in liver tissue was more scattered, and the degree of adipose degeneration in hepatocytes was improved compared with that in the HF group (Figure 3).

### 2.4. Results of 16S rRNA Sequencing of Mouse Colon Contents

**Figure 4:**
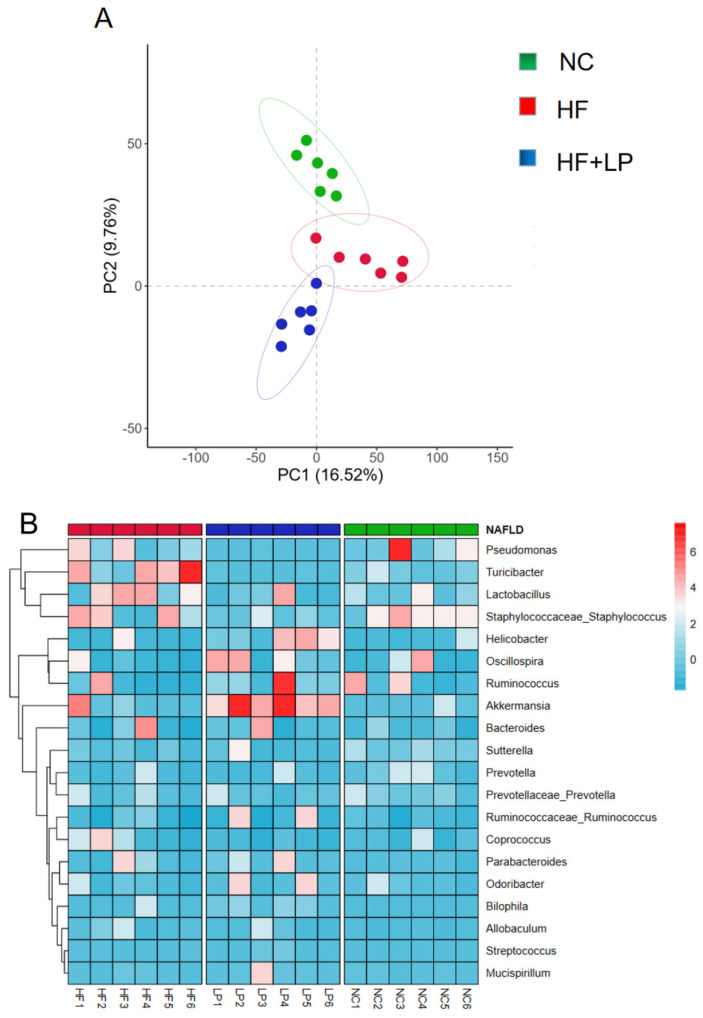
Analysis of intestinal flora 16S rRNA in mice. (**A**) PCA analysis of intestinal flora; (**B**) heat map analysis of intestinal flora.

PCA graph analysis showed complete separation of the three groups of samples. The results indicated that after had been NAFLD induced, the NC group and the HF group were significantly separated, while after the *L. plantarum* treatment, the HF + LP and HF groups were significantly separated, indicating that *L. plantarum* had a regulatory effect on the intestinal flora disorder of the NAFLD mice (Figure 4A). Compared with the NC group, the abundance of *Pseudomonas* and *Turicibacter* in the intestinal tract of the mice in the HF group was significantly increased. After treatment with *L. plantarum*, the abundance of *Pseudomonas* and *Turicibacter* decreased significantly, while the abundance of *Akkermansia* increased significantly (Figure 4B).

### 2.5. Effect of NKK20 on Metabolomics in Colon Contents

**Figure 5:**
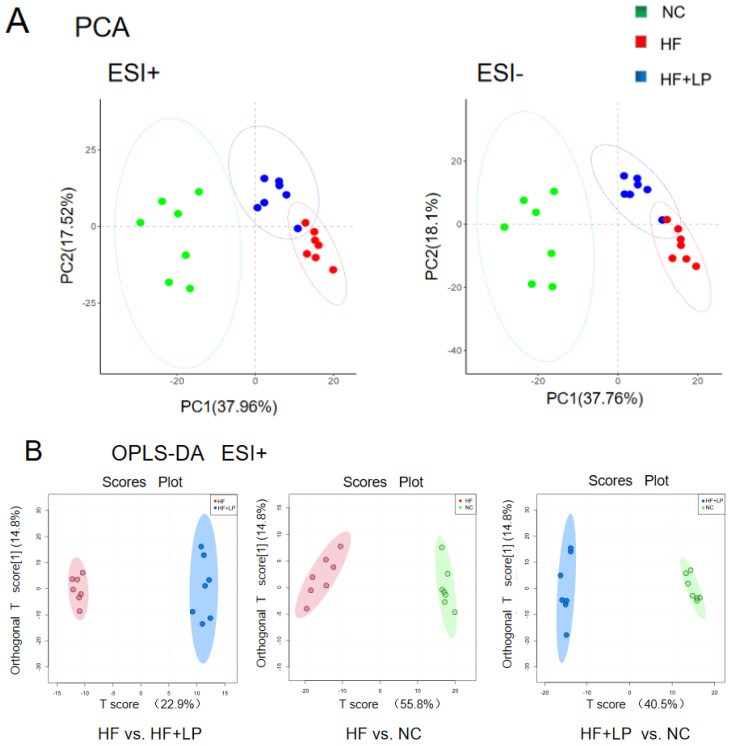

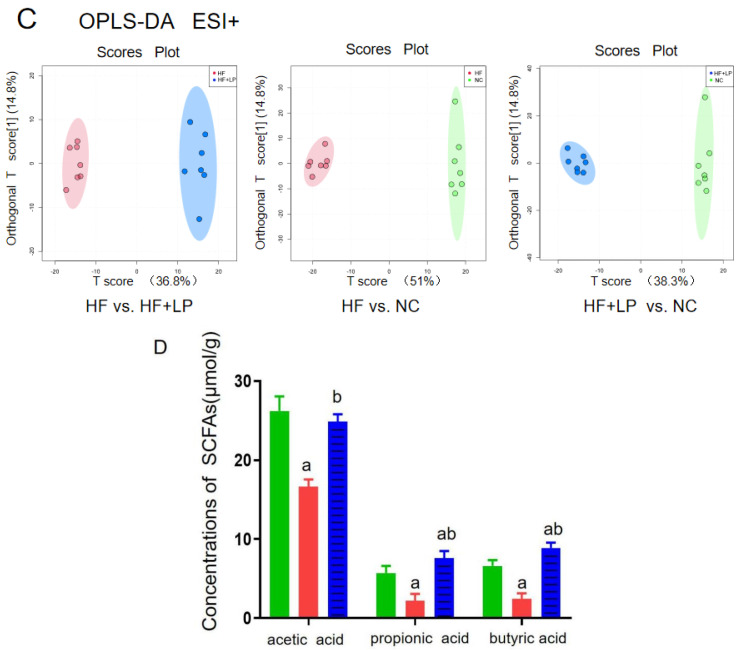
The targeted/untargeted metabolomics analysis of colon contents of mice. The untargeted metabolomics PCA model (**A**) and OPLS-DA model (**B**,**C**) analysis results; (**D**) targeting metabolomics analyzing three major SCFAs’ outcomes in colon contents. a: Compared with the NC group, *p* < 0.05; b: compared with the HF group, *p* < 0.05.

The results regarding the conducted principal component analysis (PCA) and orthogonal partial least squares discriminant analysis (OPLS-DA) of untargeted metabolomics on the colon contents are shown in Figure 5A. The OPLS-DA analysis showed that the NC, HF, and HF + LP groups were clustered and that the HF + LP group was closer to the NC group (Figure 5B,C). The OPLS-DA model parameters were R^2^X = 0.676, R^2^Y = 0.981, and Q^2^ = 0.936. This result shows that the model has good interpretive capacity and predictability. The variable importance in the projection (VIP) score reflects the contribution of the analyzed variables to the OPLS-DA model. The S-plot reflecting the contribution rate between the groups was further processed, and compounds meeting the screening conditions VIP > 1 and *p* < 0.05 in the t-test were selected as potential differential metabolites for further identification. Eleven kinds of potential differential metabolites of *L. plantarum* NKK20 were obtained using secondary mass spectrometry information on the compounds; retrieval and matching of the HMDB metabolic database and literature information are shown in Table 1.

**Figure 6:**
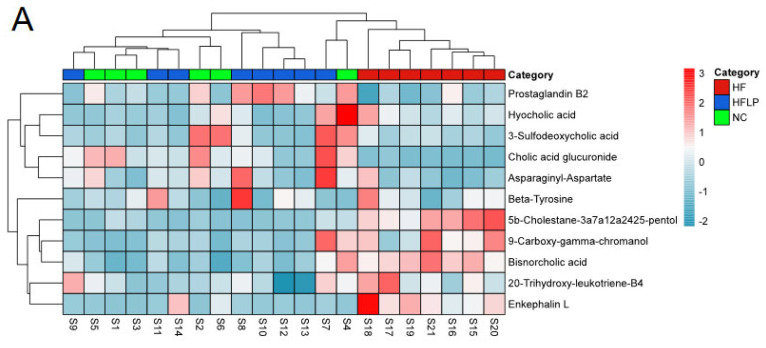

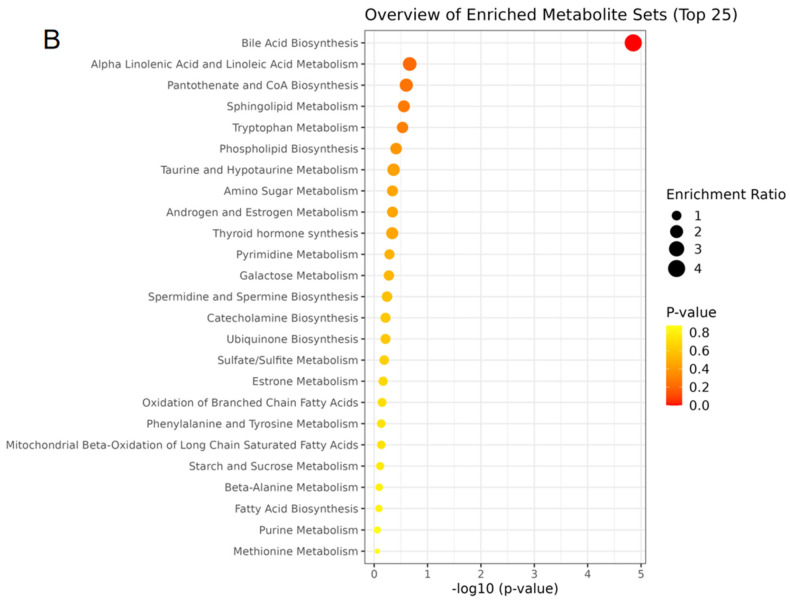
The heat maps of differential metabolites in mouse colon contents and analysis of related metabolic pathways. (**A**) The heat maps of differential metabolites in mouse colon contents; (**B**) the analysis of related metabolic pathways.

The content of the three main short-chain fatty acids (SCFAs) in the colon contents of all groups showed significant changes (Figure 5D). Compared with the NC group, the acetic acid, propionic acid, and butyric acid concentrations in the HF group were significantly decreased (*p* < 0.05), while the concentrations of propionic acid and butyric acid in the HF + LP group were significantly increased (*p* < 0.05). Compared with the HF group, the concentrations of the three main SCFAs were significantly increased (*p* < 0.05). The above differential metabolites are mainly involved in steroid metabolism, taurine metabolism, amino acid metabolism, glycerophospholipid metabolism, bile acid anabolism, etc. (Figure 6).

### 2.6. NKK20 Significantly Affected Bile Acid Metabolism in Mice

**Figure 7:**
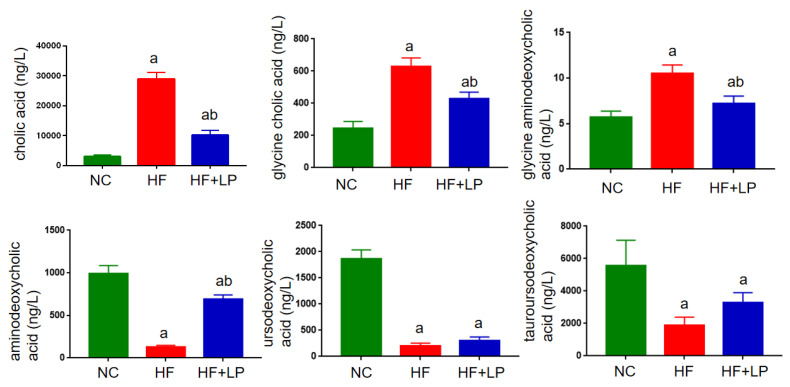
UPLC-MS/MS analysis of liver bile acid concentrations in mice. a: Compared with the NC group, *p* < 0.05; b: compared with the HF group, *p* < 0.05.

In view of the non-targeted metabolomics results suggesting that *L. plantarum* NKK20 can significantly affect bile acid metabolism in mice, the UPLC-MS/MS technique was used to determine the concentrations of six conjugated and free bile acids in mouse liver. The contents corresponding to the bile acid spectrum in the liver changed with NAFLD. The primary bile acids cholic acid and gandeoxycholic acid can be combined with taurine and glycine to form conjugated bile acids such as gandeoxycholic acid and gandeoxycholic acid. These conjugated bile acids will produce secondary bile acids, such as ursodeoxycholic acid, under the action of intestinal flora. In general, primary bile acids are highly toxic, and the accumulation of large amounts of primary bile acids can cause liver cell damage. Compared with the NC group, the concentrations of cholic acid, glycine cholic acid, and glycine aminodeoxycholic acid in the livers of the mice in the HF group were significantly increased, while the concentrations of aminodeoxycholic acid, ursodeoxycholic acid, and tauroursodeoxycholic acid were significantly decreased (*p* < 0.05). Compared with the HF group, the concentrations of cholic acid, glycinocholic acid, and glycinodeoxycholic acid in the livers of mice in the HF + LP group were significantly decreased, and the concentration of aminodeoxycholic acid in the HF + LP group was significantly increased (*p* < 0.05), while the concentrations of ursodeoxycholic acid and tauroursodeoxycholic acid were not significantly changed (*p* > 0.05) (Figure 7).

## 3. Discussion

The intestinal flora is a complex bacterial community that exists in a symbiotic partnership with the host and plays a critical role in digestion, metabolism, and host protection [15]. The relationship between the intestine and the liver is very close. A close relationship exists between the intestine and the liver since intestinal bacteria release metabolites through hepatic portal veins [16,17]. After transplanting feces from healthy donors into the intestines of patients with severe liver disease, the 1-year survival rate of patients with severe liver disease was increased by 54.2%, indicating that intestinal flora plays an important role in the occurrence and development of liver diseases [18,19]. There are also metabolites of intestinal flora that affect NAFLD development and occurrence [20,21].

Bile acids are the main metabolites of intestinal flora that facilitate the digestion and absorption of fat-soluble foods [22,23]. Intestinal flora also significantly affects the metabolism and transport of bile acids; in particular, it can directly regulate the transformation of primary bile acids to secondary bile acids. In turn, when intestinal flora is disturbed, the transformation from primary bile acid to secondary bile acid is halted, which affects the homeostasis of the bile acid pool and ultimately affects the metabolism of sugar and lipids in the human body [24,25,26]. NAFLD is closely associated with bile acid anabolism disorders [27,28]. Studies have shown that removing harmful bacteria using antibiotics and increasing the abundance of bacteria with bile saline-hydrolyzing activity in the gut can mitigate the development of NAFLD and alter the composition of the bile acid pool and the farnesoid X receptor signaling pathway [29,30]. According to these studies, the intestinal flora regulates bile acid metabolism and NAFLD outcome through glucolipid metabolism [31].

In this study, based on untargeted metabolomics results, we found that after the oral administration of *L. plantarum* NKK20, several bile acids in the colons of the studied mice were changed, including cholic acid glucuronide, hyocholic acid, bisnorcholic acid, and 3-sulfodeoxycholic acid. Hyocholic acid, bisnorcholic acid, and 3-sulfodeoxycholic acid are secondary bile acids [32]. After administration of *L. plantarum* NKK20, the concentrations of the above secondary bile acids in the colons of mice were significantly decreased. It is possible that the liver may synthesize bile acids from cholesterol when the concentrations of secondary bile acids are reduced, which helps to lower serum TC concentrations [33]. Bisnorcholic acid and 3-sulfodeoxycholic acid are free bile acids, which are toxic to intestinal mucosa in high concentrations [34]. In this study, it was found that *L. plantarum* NKK20 could reduce the concentrations of the above free bile acids in the intestinal tract, while NKK20 will prevent intestinal mucosal damage and maintain the integrity of the mucosal barrier. Endotoxins from Gram-negative bacteria in the gut can cause systemic inflammation if they enter the bloodstream through an intact intestinal mucosal barrier [35]. Bacterial endotoxins from the gut constitute a major cause of NAFLD [36]. 

5b-Cholestane-3a,7a,12a,24,25-pentol (5b-CP), a precursor of bile acid, has been shown to be significantly elevated in the gut of patients with inflammatory bowel disease, suggesting that it is associated with intestinal inflammation [37]. In our study, it was found that the concentration of 5b-CP in the intestinal contents was significantly decreased after the administration of *L. plantarum* NKK20. This result indicated that *L. plantarum* NKK20 had a potential inhibitory effect on inflammation. Prostaglandin B2, the most abundant prostaglandin released by osteoblasts, is an important metabolite of the arachidonic acid pathway [38]. Studies have shown that oral administration of probiotics (Probio-M9) can significantly increase the concentrations of SCFAs such as propionic acid, butyric acid, and isobutyric acid in the intestinal tracts of adults, thereby upregulating the metabolic pathways of compounds such as arachidonic acid, α-linolenic acid, linoleic acid, and tryptophan and increasing the concentrations of beneficial metabolites such as arachidonic acid, leukotriene B4, prostaglandin B2, and linoleic acid in the intestinal tract [39]. Additionally, the blood concentrations of these metabolites increase [40]. Our results showed that NKK20 did not effectively prevent weight gain in mice on a high-fat diet, but it did downregulate the levels of TG and TC, inhibit spleen inflammation, and increase CYP7A1 expression in the liver. CYP7A1 is a rate-limiting enzyme that catalyzes the transformation of liver cholesterol into bile acids [41]. Even though the exact mechanism of the occurrence of NAFLD is still unclear, oxidative stress; enhanced TG and TC levels; hepatic fat accumulation; the activation of nuclear factor kappa-B/NLR family and the pyrin domain-containing protein 3 (NF-κB/NLRP3) inflammasome; and the eventual formation of liver fibrosis are all factors that interact and induce synergistic effects [42]. It is important to recognize that intestinal flora and its metabolites play an important role in this process and that the ratio of cholic acid/aminodeoxycholic acid in the liver is proportional to the level of triglyceride in the liver and the degree of liver injury [43]. Targeted metabolomics technology was used to further detect six kinds of bile acids, and it was found that the ratio of cholic acid/andeoxycholic acid in NAFLD mice was significantly higher than that in the NC group, while NKK20 could significantly reduce this ratio, which was consistent with the result regarding NKK20 alleviating liver damage in mice.

SCFAs are a class of saturated fatty acids with a carbon chain of 1-6 atoms, including formic acid, acetic acid, propionic acid, butyric acid, valeric acid, caproic acid, and their isomers. In this study, after the administration of *L. plantarum* NKK20, we found that the concentrations of the three SCFAs (acetic acid, propionic acid, and butyric acid) significantly increased, while the concentrations of multiple secondary bile acids significantly decreased, which may be related to a decrease in the abundance of *Pseudomonas* and *Turicibacter* and an increase in the abundance of *Akkermansia* in mice. According to the axis principle of “intestinal microbiota—metabolite—immunity”, during gastrointestinal transit, SCFAs are metabolites of intestinal bacteria that enter the bloodstream and play an anti-inflammatory and liver-protective role [44,45]. They are produced by anaerobic bacteria in the intestinal tract that ferment dietary fiber incapable of being digested or absorbed by human beings in the colon segment [46]. SCFAs can participate in various metabolic processes, including gastrointestinal hormone secretion and fat and glucose metabolism [47]. SCFAs also regulate insulin and glucagon secretion via G-protein-coupled receptors (GPCRs). SCFAs can regulate body fat production, reduce free fatty acid levels in overweight patients, and mitigate metabolic disorders [48]. SCFAs also inhibit the activity of NF-κB by inhibiting histone deacetylase (HDAC) activity, thereby affecting the composition and function of T cells and maintaining immune homeostasis [49]. Through the inhibition of HDAC-mediated rapamycin target proteins, which are important kinases for T cell differentiation, SCFAs induce intestinal T cell differentiation and maintain balance within this process [50].

An important direction of the biological transformation of cholesterol in vivo is its degradation into bile acids [51]. If the cholesterol in the liver is not expediently transformed into bile acids, the accumulation of cholesterol and other lipids in the liver will exceed the organ’s compensatory capacity, resulting in enhanced endoplasmic reticulum stress and mitochondrial oxidative stress in the liver cells and thus activating the NLRP3 inflammasome via cholesterol crystallization, which, as a result, promotes inflammation [52]. Therefore, it is important to reduce liver fat and cholesterol accumulation. Cholesterol enters the liver mainly in the form of LDL-C. A probiotic dietary intervention that lowers cholesterol concentrations by affecting intestinal bile acids may be the key to reducing disease risk and improving health [11,12]. The probiotic L. rhamnosus GG can increase the expression of liver CYP7A1 and potentially affect the synthesis of endogenous cholesterol through the HMGR gene, thus increasing the clearance rate of circulating LDL [53]. The reduction in cholesterol transport to the liver and a reduction in liver inflammation can be achieved using *L. plantarum* NKK20. The present study has several limitations that need to be addressed in future studies. (1) The levels of bile acid profiles in the serum and intestine have not been investigated and thus require further research. (2) The gut microbiota can affect NAFLD; hence, further investigation is needed to determine whether the gut microbiota regulates bile acid anabolism in NAFLD mice.

## 4. Materials and Methods

### 4.1. NKK20 Preparation

*Lactiplantibacillus plantarum* NKK20 specimens were obtained from Zhenjiang Tianyi Biotechnology Co., Ltd. (Zhenjiang, China, China Typical Culture Storage Center, Storage number CCTCC NO: M2020596). The freeze-dried bacterial powder was dissolved with appropriate amount of sterilized normal saline to prepare the suspension as described by Bi et al. [54]. Colony counting was conducted before the animal experiments to adjust the number of surviving bacteria to 5 × 10^9^ CFU·mL^−1^.

### 4.2. Animals Diet and Treatment

The male ICR mice (32 g ± 4 g) were purchased from the Comparative Medical Center of Yangzhou University (Yangzhou, China) and raised in the barrier system of the Laboratory Animal Center of Jiangsu University. After a week of adaptation, the ICR mice were randomly divided into 3 groups with 10 mice in each group, namely, a normal control group (NC group), a high-fat group (HF group), and an *L. plantarum* NKK20 intervention group (HF + LP group). From the beginning of the experiment, mice in the HF group and the HF + LP group were fed with a high-fat diet (containing 60% fat for energy) developed by Nanjing Synergetic Biology Company (Nanjing, China) (Table 2). The caloric value of the normal diet was 3.84 kcal/g, and that of the high-fat diet was 5.24 kcal/g. Changes in average body weight (g), average feed quality (g), and average energy intake (kcal) of each mouse in each group were monitored throughout the experiment.

Starting on the fifth week, the mice of the HF + LP group were given *L. plantarum* intragastrically with 1 × 10^9^ CFU/animal/day until the end of the experiment on the 12th week. The mice in each group were given sterilized purified water to drink freely, and the mice in NC group were given conventional feed. Mice were anesthetized via intraperitoneal injection of Escloamdone. Then, blood was taken from the posterior orbital venous plexus of mice, and the plasma was isolated and stored in the refrigerator at −80 °C after anticoagulation with heparin sodium. Mouse spleens and liver colon contents were kept in a −80 °C refrigerator.

### 4.3. Plasma Parameters

The TG, TC, high-density lipoprotein cholesterol (HDL-C), and low-density lipoprotein cholesterol (LDL-C) levels in the serum were measured using the purchased commercial kits from Nanjing Jiancheng Bioengineering Institute (Nanjing, China) in accordance with the manufacturers’ instructions.

### 4.4. RNA Extraction and qPCR Assay

The experimental methods employed were based on those reported by Feng et al. [55], and the experimental process is as follows. Total RNA was extracted from mouse livers and spleens via the Trizol method and reverse-transcribed into cDNA (Vazyme, Nanjing, China). The qRT-PCR assay was used to determine the mRNA expressions of inflammatory factors in spleen and the mRNA expression of enzymes related to bile acid metabolism in liver. The total volume of qRT-PCR was 20 μL, including SYBR Green Master premix (Vazyme, Nanjing, China) at a volume of 10 μL, upstream and downstream primers at a volume of 0.4 μL (10 μmol/L), and cDNA template at a volume of 2 μL. The reaction procedure included pre-denaturation at 95 °C for 5 min, denaturation at 95 °C for 3 s, annealing at 58 °C for 20 s, extension at 72 °C for 30 s, and a total of 40 reaction cycles. The mouse actin gene was used as the internal reference to calculate the mRNA relative expression using the following formula: 2^−ΔΔCt^. The primer sequences used, which were synthesized by GENEWIZ (Suzhou, China), are shown in Table 3.

### 4.5. Liver Histopathological Observation

In this study, hematoxylin and eosin (H&E) staining was employed according to Xi et al.’s method, which was modified slightly [56]. The liver tissues of mice were immersed in 4% polymethanol and fixed. The liver tissues were stained with H&E in the Department of Pathology in Affiliated Hospital of Jiangsu University (Zhenjiang, China). The liver tissues were observed under an optical microscope and recorded photographically.

### 4.6. Analysis of Intestinal Flora of Mice

Colon contents of mice were collected, and intestinal microbial genomic DNA was extracted using a genomic DNA extraction kit (TIANGEN, Beijing, China) for intestinal flora analysis. Intestinal flora analysis was performed by Wekemo Tech Group Co., Ltd. (Shenzhen, China). The high-throughput sequencing platform IlluminaHiseq was used for sequencing, and biological information analysis was used to conduct sequence analysis and species annotation. Through OTU cluster analysis, community composition and structure of each group of samples at different levels were obtained, alpha diversity was analyzed, and differences among samples were compared.

### 4.7. Untargeted/Targeted Metabolomics Analysis

In this study, the untargeted metabolomics analysis and the determination of the three main SCFAs in mouse plasma were conducted according to Fen et al.’s report [55]. A total of 50 mg of colon contents and 400 μL of extract solution (methanol: double steaming water = 4:1) were mixed, and the mixture was crushed on ice with a tissue crusher. The mixture was placed at −20 °C for 30 min and centrifuged at 16,400× *g* for 15 min. The supernatant was used for non-targeted metabolomics analysis via UPLC-QTof-MS/MS system in the Test Center of Yangzhou University, and the concentrations of three main SCFAs (acetic acid, propionic acid and butyric acid) were analyzed using an LC-MS/MS system. Kyoko Encyclopedia of Genes and Genomes (KEGG) database (https://www.genome.jp/kegg/pathway.html, accessed on 10 October 2022) was used to analyze the differential metabolites involved in metabolic pathways. For multivariate statistical analysis, principal component analysis (PCA) and partial least squares discriminant analysis (PLS-DA) were used to obtain the VIP value of each metabolite using the metabonomics data-processing software metaX. Univariate analysis was performed using *t*-test to analyze the significant differences between the metabolites in all groups (*p* value). The screening parameters for differential metabolites were VIP > 1.0 and *p* < 0.05.

### 4.8. Liver Bile Acid of Mice Was Detected by UPLC-MS/MS

A total of 50 mg of liver tissue was combined with 1mL of methanol homogenate and centrifuged at 4 °C for 10 min at 16,000× *g*. The supernatant, with a volume of 800 μL, was dried with nitrogen and then redissolved with 100 μL of 70% methanol for UPLC-MS/MS analysis. The UPLC-MS/MS analysis process was conducted as follows. Separation was performed on Acquity UPLC^®^ BEH C18 column (100 mm × 2.1 mm, 1.7 µm) using Dionex Ultimate 3000 High-pressure liquid system (ThermoFish, Waltham, MA, USA). The mobile phase was 5 mmol/L ammonium acetate containing 0.1% formic acid (A) and methanol (B). Gradient elution procedure: 0–1 min (55% B), 1–2.6 min (55–62% B), 2.6–11.4 min and (62–80% B). The flow rate was 0.30 mL/min, the sample size was 5 μL, and the column temperature was 45 °C. Q Exactive quadrupole/electrostatic field orbital trap high-resolution mass spectrometer was used for analysis and detection. Ion source: electrospray ion source (H-ESI); negative ion mode full-scanning-single-ion-monitoring (scan-SIM) mode was used with the following data mass spectrum parameters: scanning range (100–1500 *m/z*); resolution 70,000; spray voltage 2.8 kV; capillary temperature 320 °C; auxiliary heater temperature 300 °C; sheath gas (N_2_) flow 35 arb; auxiliary gas (N_2_) flow rate 13 arb; automatic gain control 10^6^; s-lens RF Level 50.

### 4.9. Statistical Analysis

Data are presented as means ± standard errors (SD). Differences between groups were examined through one-way analysis of variance, with post hoc analysis conducted using either Tukey’s test for multiple comparisons according to the result of Bartlett’s test for equal variances by using the GraphPad software (Prism, San Diego, CA, USA).

## 5. Conclusions

**Figure 8:**
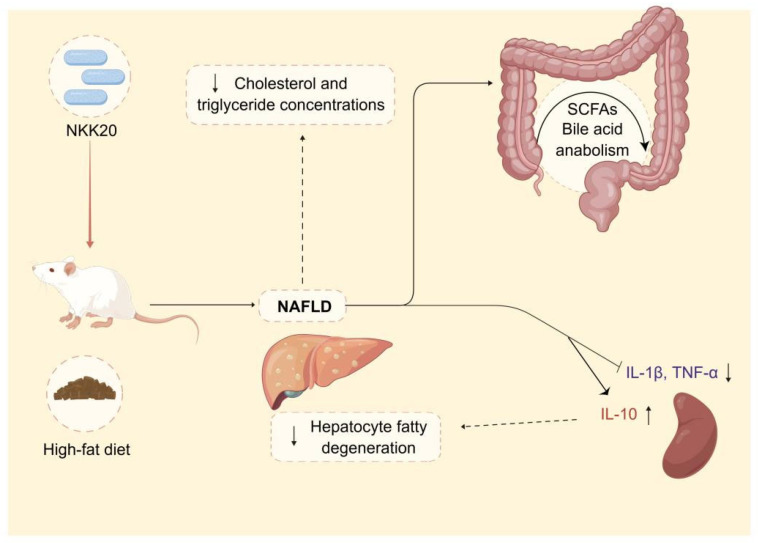
The potential therapeutic effect and mechanism of *L. plantarum* NKK20 with respect to NAFLD. *L. plantarum* NKK20 alleviates inflammatory liver injury through reducing liver damage and systemic inflammation, reducing blood triglyceride and TC concentrations, promoting secondary bile acid reabsorption, and regulating bile acid anabolism in high-fat-diet-induced NAFLD mice.

In summary, *L. plantarum* NKK20 showed characteristics of probiotics and reduced liver damage, systemic inflammation, and blood triglyceride and TC concentrations in an NAFLD murine model. Moreover, *L. plantarum* NKK20 suppressed lipid accumulation in the livers of the mice by increasing the expression of CYP7A1. Furthermore, bile acid anabolism was altered, and the concentrations of SCFAs were increased. The results of this study provide a theoretical basis for the treatment of NAFLD with *L. plantarum* NKK20 based on the “intestinal microbiota—metabolite—inflammation” axis (Figure 8). Therefore, *L. plantarum* NKK20 may be used as a potential probiotic for the treatment and prevention of NAFLD.

## Figures and Tables

**Table 1:** The different metabolites in colonic contents of mice in HF + LP group compared with HF group.

Metabolites	Related Metabolic Pathways	*m/z*	Retention Time (min)	VIP	*p* Value	Change Trend
Cholic acid glucuronide	Bile acid biosynthesis	619.2886636	4.50925	1.48	0.010	↑
Asparaginyl-Aspartate	Amino acid metabolism	493.1515701	13.59033333	1.42	0.010	↑
Tyrosine	Amino acid metabolism	180.066612	1.814466667	1.40	0.010	↑
20-trihydroxy-leukotriene-B4	Steroid metabolism	365.1966849	4.9492	1.391	0.000	↑
Enkephalin L	Steroid metabolism	554.2620907	22.95986667	1.37	0.000	↑
9′-Carboxy-gamma-chromanol	Bile acid biosynthesis	397.2362352	16.50585	1.16	0.001	↓
Prostaglandin B2	Arachidonic acid metabolism	461.1969152	4.7223	1.15	0.001	↑
Hyocholic acid	Bile acid biosynthesis	389.2697488	5.430083333	1.14	0.001	↓
5b-Cholestane-3a,7a,12a,24,25-pentol	Bile acid biosynthesis	433.3323119	8.048866667	1.15	0.024	↓
Bisnorcholic acid	Bile acid biosynthesis	361.2387639	5.869866667	1.22	0.000	↓
3-Sulfodeoxycholic acid	Bile acid biosynthesis	439.2160426	7.62975	1.17	0.000	↓

Note: ↑ and ↓ indicate increase and decrease, respectively, vs. HF group.

**Table 2:** Feed energy supply of mice.

Components	Normal Diet (kcal/gm)	High-Fat Diet (kcal/gm)
Casein	800/200	800/200
L-Cystine	12/3	12/3
Corn Starch	2024.8/506.2	0/0
Maltodextrin	500/125	500/125
Sucrose	275/68.8	275/68.8
Cellulose	0/50	0/50
Soybean Oil	225/25	225/25
Lard	180/20	2205/245
Mineral Mix	0/10	0/10
DiCalcium Phosphate	0/13	0/13
Calcium Carbonate	0/5.5	0/5.5
Potassium Citrate	0/16.5	0/16.5
Vitamin Mix	40/10	40/10
Choline Bitartrate	0/2	0/2
Total kcal/g	4056.8 kcal/1055 gm = 3.84	4057 kcal/773.8 gm = 5.24
Nutrient composition	Energy supply ratio	
Protein	20%	20%
Carbohydrate	70%	20%
Fat	10%	60%

**Table 3:** The qRT-PCR primer sequences.

Genes	Primers Sequences (5′→3′)
*β-actin*	F: AAGCTGTGCTATGTTGCTCTA
	R: GTTTCATGGATGCCACAGGA
*TNF-α*	F: AACTCCAGGCGGTGCCTATG R: TCCAGCTGCTCCTCCACTTG
*IL-1β*	F: CCTGTCCTGCGTGTTGAAAGA R: GGGAACTGGGCAGACTCAAA
*IL-4*	F: CCATATCCACGGATGCGACA R: AAGCACCTTGGAAGCCCTAC
*IL-10*	F: GAAGCTCCCTCAGCGAGGACA R: TTGGGCCAGTGAGTGAAAGGG
*CYP7A1*	F: CCGATGGATGGAAATACCAC R: GGCAGCGGTCTTTGAGTTAG
*ASBT*	F: TACGGTAGCAGGGGTTTACG R: TGCAAAATGGAACAAAACCA
*LDLR*	F: GAATTTGGCCAGACACAGGT R: CACCGTACCCAGCTGATTTT
*HMGR*	F: GTCATTCCAGCCAAGGTTGT R: GGGACCACTTGCTTCCATTA
*TGF-β1*	F: GTCAGACATTCGGGAAGCAG
	R: GCGTATCAGTGGGGGTCA
*CTGF*	F: CAACCGCAAGATTGGAGTGT
	R: CTCCAGTCTGCAGAAGGTATTG

## Data Availability

The data presented in this study are available on request from the corresponding authors.
